# Communities as co-producers in integrated care

**DOI:** 10.5334/ijic.1589

**Published:** 2014-06-30

**Authors:** Henk Nies

**Affiliations:** Of the Executive Board, Vilans, Utrecht; Organisation and Policy in Long-term Care, VU University of Amsterdam, Amsterdam, The Netherlands

**Keywords:** integrated care, community, co-producer, professional

## Abstract

Integrated care has become too much a professionals' concept, in research and theory development, as well as in practice, especially in high-income countries. The current debate on integrated care is dominated by norms and values of professionals, while most of the care is provided by non-professionals. The paradigms of integrated care for people with complex needs need to be reconsidered. It is argued that non-professional care and care by local communities need to be incorporated as a resource and a co-producer of care. It seems fair to assume that the community as such can take a more prominent role in organising and delivering health and long-term care. This implies redefining professional and non-professional responsibilities and boundaries. The boundary between public and private space is losing its significance, as is the distinction between formal and non-formal care. It also requires renegotiating and transforming organisational boundaries. This has consequences for legislation, funding and professional qualifications, as well as for management and governance. It challenges current professional identities as well as identities of service users, their informal carers and citizens. It may also require new types of funding, including non-monetary currencies, time-sharing and social impact bonds. The challenge is that big, that it needs to be addressed at its smallest scale: the citizen in his social network and local community, being co-producer of really integrated care.

## Introduction

Being involved in integrated care at local, regional, national and international levels for more than 20 years, I have come to the conclusion that this field has become too much professionalised in high-income Western countries. Although it is a young branch of health services research, it mirrors itself too much to established practices in ‘traditional’, non-integrated health care. The basic paradigms of integrated care need to be reconsidered in order to make a significant contribution to health and well-being of people with complex needs. Complex needs require answers that are often provided by complex and fluid organisational structures, meeting the idiosyncrasy and dynamics of the person (by professionals often referred to as: ‘service user’, ‘patient’ and ‘client’). These organisational structures should consist of professional and non-professional people who provide care, treatment and support. In fact, they very often do consist of professional and non-professional care providers, but usually they do not function as well as integrated systems.

The issue is that the current debate on integrated care is a professional debate. It is very much dominated by norms and values of professionals. It looks as if care provision is only a responsibility of professionals, whereas about 75–80% of all care is provided by informal carers [1, 2]. Informal care is an enormous source of care, especially in long-term care.

The current body of knowledge on integrated care primarily focuses on professional service integration at the clinical (or service), professional, organisational and system levels [e.g. 3–7]. Integrated care is traditionally conceived as a form of inter-professional and inter-organisational collaboration, where integration can be horizontal and vertical [e.g. 8, 9].

But is this the full picture? In my view, we need to include non-professional and community resources and structures as co-producers of health and social care in order to achieve sustainable care. This implies that we need to reconceptualise integrated care to include the community as a resource and a co-producer of care. This holds in particular for people with long-term complex and multiple needs. In addition to this reality of care provision, there are at least three other good reasons to justify this view.

## Changing concept of health

First, the current concept of health as defined by the WHO in 1948 appears to be outdated. As Huber et al. [10] argue, the conceptualisation of health as ‘a state of complete physical, mental and social well-being and not merely the absence of disease or infirmity’ in fact declares the vast majority of our population as unhealthy, with over-medicalisation as the obvious risk. ‘It minimises the role of the human capacity to cope autonomously with life's ever-changing physical, emotional and social challenges and to function with fulfilment and a feeling of well-being with a chronic disease or disability’ [10, p. 236].

Ageing with multiple chronic illnesses becomes the norm for most people. As life expectancy is increasing in most countries, life expectancy without illness is decreasing [11,12]. According to the new definition, ‘health’ should be conceived as the ‘ability to adapt and self manage in the face of social, physical, and emotional challenges’ [10, p. 235]. Our twenty-first century society with proper housing conditions, opportunities for good nutrition, healthy life styles, high levels of education, social security and (assistive) technology enables compensation mechanisms more than ever before.

Therefore, integrated care for people with complex needs should aim at strengthening coping capabilities, resilience and supportive conditions in the physical, mental and social domain to enable an (adapted) equilibrium [10, 13]. Therefore, the solution to health problems and impairments often lies outside the realm of professional health care and in the community or in society. In daily life, the community or society consists of kin, neighbours, volunteers, other people in the neighbourhood, local associations, churches, firms, etc. Integrated care to people with complex needs should therefore aim at a supportive environment. And when this equilibrium is achieved, they do not consider themselves as a ‘patient’, but as a ‘citizen’, member of a community, or as a person [13, 14]. This community is not a passive condition, it often is an actor, that actively engages, or a resource for strengthening the individual's equilibrium.

## Ecological approaches

Second, it is quality of life that counts, rather than quality of care as such. This concept relates to independence, autonomy, participation, personal fulfilment and dignity [15, 16]. Schalock and Verdugo [17] argued that quality of life in people with mental retardation is associated with emotional well-being, interpersonal relationships, material well-being, personal development, physical well-being, self-determination, social inclusion and rights. It seems fair to assume that these domains are also relevant to other categories of people with long-standing, complex conditions, such as frail older people [18]. In fact, these categories apply to all people, irrespective disease or impairment. Quality of life, in its full range, implies a holistic approach [10].

If this is the case, we need more ecological approaches. Schalock and Verdugo [17] propose a distinction of micro-, meso- and macro-systems. The first – the micro-system – is the immediate social setting, such as the family, peers and the work-setting, people who directly affect the person's life. The second – the meso-system – refers to the neighbourhood, the community, service organisations and other agencies that directly affect the micro-system. The third – macro – system is the overall pattern of culture, socio-political trends, the economic system and society-related factors that affect people's lives. In other words, in order to be effective, health care professionals and organisations should operate in a person's ecology in order to be relevant to quality of life. They are, in fact, part of this ecology, not a separated entity. So engaging the community in which people live is in fact working in and with people's ecology to support or even strengthen their coping capabilities and resilience.

## Populations taking the lead

Third, there is growing interest in population- and area-oriented strategies. These usually imply developing integrated service delivery for specific populations, but without taking these populations themselves as an actor or co-producer of health and well-being. This interest goes hand-in-hand with discussions on allocating funding based on outcomes at population level [6, 19]. In fact, we see services and policy-makers trying to manage populations or communities with a preference to use terms as ‘population management’, without questioning themselves, how populations or communities can manage services and policy-makers.

In my own country, the Netherlands, we see a rapidly increasing interest in citizens’ initiatives, organising and purchasing their own care and support. Local communities – or populations – take the lead and develop themselves according to cooperative principles. This trend reflects the discontent with existing services, but also concerns for the quality of the local community and taking responsibility [e.g. 20]. And recently, where care homes are being closed because of budget cuts by central government, we see small communities taking action and considering adopting some of these homes.

## Consequences for integrated care

In the conceptualisation of integrated care, we should incorporate communities and their members as co-producers of health and well-being. In high-income countries, care for people with complex needs is often seen as a specialised professional and institutionalised (or highly standardised) domain. In terms of organisational responsibilities, it is strictly separated from the informal domain and from the community. In fact, we have organised professional care outside the community. This segregation has led to alienation and disengagement of citizens from care.

It seems fair to assume that the community as such can take a more prominent role in organising and delivering health and long-term care. There are examples of community engagement from other countries such as Finland, Denmark and the UK. Follow-up of these examples in which communities are actually co-producing, instead of being an object of an intervention are scarce [21, 22]. Moreover, they are not incorporated in our theorising on integrated care.

If we do so, we need to redefine professional and non-professional responsibilities and boundaries. Organisational boundaries are moving, dependent of personal, social, organisational and local resources; the available capabilities and conditions. Fluid organisational structures with blurring boundaries are emerging. They are hard to align with traditional principles of governance in health care with fixed boundaries. Existing agreements on professional responsibilities, standards, guidelines, privacy regulations, payments, etc. need to be renegotiated and new uncertainties of professionals and non-formal actors need to be dealt with. Integration should be in fact conceived as redefining boundaries, instead of ‘difference management’ as Gobet and Emilsson [23] typify it.

## Conclusions

In order to provide personalised care to people with complex needs, professional care providers need to share responsibilities with service users, their informal carers and local communities. The boundary between public and private space is losing its significance [23], as is the distinction between formal and non-formal. It requires renegotiating or – better – transforming organisational boundaries and opening up these boundaries. It has consequences for legislation, funding and professional qualifications, as well as for management and governance. It also challenges current professional identities as well as identities of service users, their informal carers and citizens.

The seeming paradox is, that health care organisations and professionals can take a lead in these processes. If they understand how sharing responsibilities works and how a concept such as ‘engaging the community’ works for people in the neighbourhood, we have a lot to win. It may also require new types of funding, including non-monetary currencies, time-sharing and social impact bonds.

Hierarchical, rule-based ways of organising need to be replaced by more fluid, network-like and consensus-based ways of working. It will often be associated with ‘messy’ issues that do not fit current organisational or professional practices [24].

The challenge of care for people with complex needs is that big, that we need to address it at its smallest scale: the citizen in his social network and local community, being co-producers of really integrated care.

## About the author

Henk Nies, PhD, is member of the Executive Board of Vilans, the Netherlands Centre of Expertise for Long-term Care. He is also professor of Organisation and Policy Development in Long-term Care at VU University in Amsterdam. Throughout his career he has worked between the boundaries of policy, practice and research. His primary field of interest is care for older people, and in particular issues regarding inter-organisational collaboration, quality management and policy development. Henk Nies has worked in several international projects, amongst others as scientific director in CARMEN, a FP5 project of the European Commission, and as a member of the Scientific Management Team of INTERLINKS, a FP7 programme. Henk Nies has published several books and many articles and blogs, in the Netherlands and abroad.
